# The Tissue-Sparing Effect of Spatially Fractionated X-rays for Maintaining Spermatogenesis: A Radiobiological Approach for the Preservation of Male Fertility after Radiotherapy

**DOI:** 10.3390/jcm9041089

**Published:** 2020-04-11

**Authors:** Hisanori Fukunaga, Kiichi Kaminaga, Takuya Sato, Ritsuko Watanabe, Takehiko Ogawa, Akinari Yokoya, Kevin M. Prise

**Affiliations:** 1 Patrick G Johnston Centre for Cancer Research, Queen’s University Belfast, 97 Lisburn Road, Belfast BT9 7AE, UK; 2 Department of Radiation Oncology, Shonan Kamakura General Hospital, 1370-1 Okamoto, Kamakura, Kanagawa 247-8533, Japan; 3 Institute for Quantum Life Science, National Institutes for Quantum and Radiological Science and Technology (QST), 2-4 Shirakata, Tokai, Ibaraki 319-1106, Japan; 4 Institute of Molecular Medicine and Life Science, Yokohama City University Association of Medical Science, 3-9 Fukuura, Kanazawa-ku, Yokohama 236-0004, Japan

**Keywords:** ex vivo model, male infertility, radiotherapy, spermatogenesis, tissue-sparing effect, tissue culture, X-rays

## Abstract

Radiotherapy can result in temporary or permanent gonadal toxicity in male cancer patients despite the high precision and accuracy of modern radiation treatment techniques. Previous radiobiological studies have shown an effective tissue-sparing response in various tissue types and species following exposure to spatially fractionated radiation. In the present study, we used an ex vivo mouse testicular tissue culture model and a conventional X-ray irradiation device to evaluate the tissue-sparing effect (TSE) of spatially fractionated X-rays for the protection of male fertility from radiotherapy-related adverse effects. We revealed a significant TSE for maintaining spermatogenesis in the ex vivo testes model following spatially fractionated X-ray irradiation. Moreover, we experimentally propose a possible mechanism by which the migration of spermatogonial cells, from the non-irradiated areas to the irradiated ones, in irradiated testicular tissue, is essential for the TSE and maintaining spermatogenesis. Therefore, our findings demonstrate that the control of TSE following spatially fractionated X-rays in the testes has a considerable potential for clinical application. Interdisciplinary research will be essential for further expanding the applicability of this method as an approach for the preservation of male fertility during or after radiotherapy.

## 1. Introduction

The protection of normal tissue, as well as the eradication of tumor, is an ultimate goal of radiation treatment. However, although radiotherapy has significantly evolved to have a high level of precision and accuracy, it can still result in temporary, long-term, or permanent gonadal toxicity in male patients [1,2]. In fact, spermatogenesis can be damaged by either direct testicular irradiation or the scattered doses received during the radiation treatment of cancers, such as prostate, bladder, rectal, and bone cancers [3]. Total doses to the testes exceeding 2.5 Gy that are delivered as multiple fractions generally produce permanent azoospermia, whereas single-fraction exposure requires doses greater than 6 Gy to produce the same effect [4,5]. The approach for the preservation of male fertility is important for radiation treatment to improve the quality of life of cancer survivors.

Tissue-level responses remarkably differ depending on whether radiation is delivered uniformly or non-uniformly [6]. In 1909, Alban Köhler reported the clinical observations of the tissue-sparing response during grid radiotherapy, in which spatially fractionated radiation is delivered using a grid-like pattern of beams [7,8]. Since the establishment of the fundamental concept of microbeam radiotherapy (MRT) in the 1990s, which is based on the spatial fractionation of synchrotron-generated X-ray microbeams at the microscale level [9], notable tissue-sparing effects (TSEs) following exposure to micro-slit X-ray microbeams have been confirmed in various species and tissue types [10,11,12,13,14,15,16]. Therefore, although the underlying mechanisms of TSE remain unclear, we hypothesize that the TSE of spatially fractionated X-rays on the testes would help preserve male fertility while still delivering high doses of radiation to the tumor.

To verify our hypothesis, in the present study, we used a conventional X-ray source and a unique ex vivo testicular tissue culture, which enabled us to monitor the progress of spermatogenesis and easily assess the radiation-induced biological effects. In addition, we used a transgenic mouse model expressing acrosome-green fluorescent protein (Acr-GFP), which is a meiosis-specific biomarker [17,18]. As previously reported, our ex vivo model of spermatogenesis can reproduce the deterministic effects of radiation (e.g., temporary infertility and permanent sterility) using a conventional 150 kVp X-ray source [19]. Furthermore, using a combination of 5.35 keV synchrotron-generated X-ray microbeams [20] and the ex vivo testes organ culture [21], we revealed, for the first time, an MRT-mediated TSE for the preservation of spermatogenesis [16]. Our experimental procedure, which used low-energy (5.35 keV) X-rays, enabled the separate investigation of the responses of the irradiated and non-irradiated areas in the tissue [22]. However, conventional X-rays and synchrotron-generated monochromatic X-ray microbeams have different energies. Thus, the TSE of spatially fractionated conventional X-rays needed immediate verification to expand the applicability of this approach.

## 2. Materials and Methods

### 2.1. Animal Model

The Acr-GFP transgenic mice were obtained from RIKEN BRC, Tsukuba, Japan, through the National BioResource Project of MEXT, Japan. These mice expressed the GFP marker specific for meiosis, which is useful for monitoring the progress of spermatogenesis [17,18]. All animal experiments conformed to the Guide for the Care and Use of Laboratory Animals and were approved by the Institutional Committee of Laboratory Animal Experimentation (F-A-17-072).

### 2.2. Ex Vivo Testicular Tissue Culture

As previously reported [19], the ex vivo testicular tissue culture method, which was developed in 2011 to produce fully functional sperm in vitro [23], allows clear and easy microscopic monitoring of spermatogenesis for more than 40 days. Testes samples were obtained from the Acr-GFP transgenic mice at around 7 days postpartum (dpp), and each sample was cut into 8–10 tissue pieces measuring approximately 1 mm^3^ in size [21]. Each tissue piece was immediately placed on a 1.5% agarose gel block immersed in 0.5 mL of αMEM containing 10% KnockOut Serum Replacement (KSR) (Gibco KnockOut Serum Replacement, Thermo Fisher Scientific K.K., Yokohama, Japan), 1% antibiotic mixture solution (Antibiotic-Antimycotic, Thermo Fisher Scientific K.K.), and 0.2 mM NaHCO_3_ in a 12-well culture dish.

The samples were cultured in a humidified incubator at 34 °C under an atmosphere of 95% air and 5% CO_2_. The Acr-GFP expressions in the culture tissue showed the progression of spermatogenesis, and in the non-irradiated cultures, the peak expression occurred at around 18–22 dpp [19]. The expression of Acr-GFP along the seminiferous tubules was designated as a sign of spermatogenesis. As previously reported [23], we confirmed that after the observation of Acr-GFP expression and the long-term culture of testicular tissue (i.e., more than 30 days), ex vivo spermatogenesis reached a mature stage and spermatogonial stem cells were differentiated to functional sperm cells and/or spermatids.

### 2.3. Live-Tissue Imaging

As stated in a past work [16,19], the cultured samples were observed by fluorescence microscopy (BZ-X700, KEYENCE) with a x4 magnification objective lens to capture GFP-fluorescent and bright-field images daily for more than 30 days. After being observed at different time points, the samples were returned to the incubator, and images were captured on alternate days. The exposure times were 1.5 s for the GFP-fluorescent images and 150 ms for the bright-field images.

### 2.4. Evaluation of Acr-GFP Expression

In accordance with a previous research [16], the Acr-GFP expression was classified into six grades, 0–5, based on the expression area: 0%, −20%, −40%, −60%, −80%, and −100%. The central area was omitted from the evaluation because this area lacked expression in many cases due to spatial and nutrient flow restrictions [23]. This issue is a technical limitation of the ex vivo testicular tissue culture method of spermatogenesis monitoring.

### 2.5. X-ray Settings

As previously reported [19], irradiation was performed using a conventional X-ray source (W150, SOFTEX, Sagamihara, Japan) operated at a tube voltage of 150 keV and a tube current of 4.1 mA. The characteristic X-rays from the tungsten anode were used to expose the samples, and the most intense energy was approximately 60 keV. An 0.2 mm aluminum filter was used to filter out X-rays lower than 7 keV. The tissues on the agarose gel in the culture dish were placed in the irradiation chamber of the X-ray apparatus and exposed to X-rays vertically through the dish lid.

The radiation-induced effects on spermatogenesis were observed by irradiating the cultured testicular tissues at 7 dpp with doses of 0.5 Gy (*n* = 4), 1 Gy (*n* = 4), 2 Gy (*n* = 4) and 5 Gy (*n* = 4) at a dose rate of 1 Gy/min or by leaving them non-irradiated (*n* = 4). To investigate the fractionated X-ray-induced effects on spermatogenesis, we also used 1 Gy × 2 (1 Gy per 24 h, *n* = 4) and 0.67 Gy × 3 (0.67 Gy per 24 h, *n* = 4). A single tissue sample from each mouse testes was randomly assigned to an experimental group. At least four tissue samples each from different donor mice were used for each experiment; contaminated samples were excluded.

**Figure 1:**
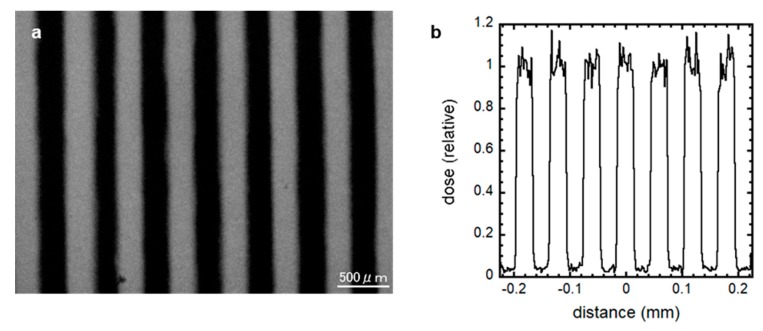
300 μm-slit-modulated beam dose profile. The modulated X-ray beams were created by shielding 50% of the cell culture using a 300 μm-slit Pb shielding material, and the dose distribution was confirmed using a Gafchromic XR-RV3 radiochromic film (**a**) and PHITS code ver. 2.96 (**b**). The irradiated regions in this optical image are shown as a black striped pattern. Scale bar, 500 μm.

Then, 3 × 15 × 0.3 mm Pb sheets (Nirako, Tokyo, Japan) and polystyrene plates through which X-rays are transmitted were alternately layered and set downstream of the X-ray source to make the 300 μm-slit-modulated X-ray beams (Figure 1). The micro-slit-modulated dose distribution was confirmed using a Gafchromic XR-RV3 radiochromic film (Ashland Inc., Covington, KY, USA). The dose profile was also calculated and confirmed by Monte Carlo simulation using PHITS code ver. 2.96 [24]. To observe the TSE of spatially fractionated conventional X-rays for preserving spermatogenesis, we irradiated the cultured samples at 7 dpp with 50% micro-slit exposure to 10 Gy (*n* = 4), equivalent to 5 Gy at the whole tissue level.

### 2.6. Statistical Analysis

One-way analysis of variance (ANOVA) was used to assess the differences in the GFP expression grades. Values with *p* < 0.05 indicated a significant difference.

## 3. Results and Discussion

### 3.1. X-ray-Induced Effects on Spermatogenesis

**Figure 2:**
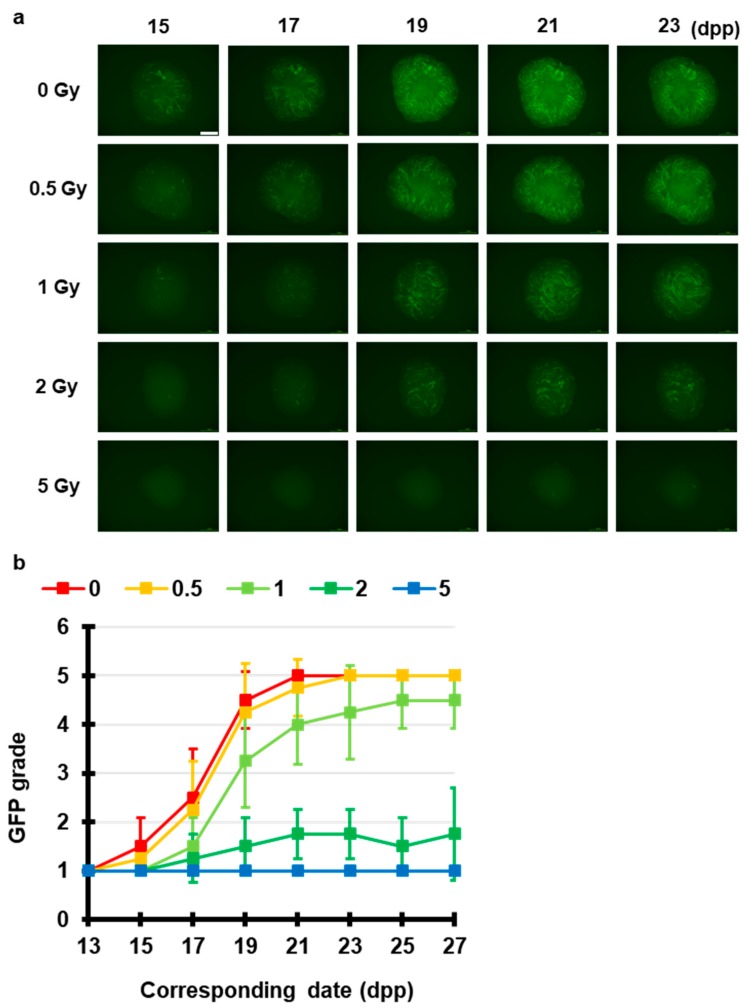
Chronological Acr-GFP expression changes after exposure to X-rays. (**a**) Representative images show Acr-GFP expression obtained at 15–23 dpp in single cultures following exposure to 0 (control), 0.5, 1, 2, and 5 Gy conventional X-rays (150 keV). Scale bar, 500 μm. (**b**) Evaluation of Acr-GFP expression from 13 to 27 dpp. Single tissue samples from each mouse testes were randomly assigned to experimental groups. At least four tissue samples each from different donor mice were used for each experiment; contaminated samples were excluded. Data show the mean Acr-GFP expression ± SD. There was statistically significant difference between group means (*p* < 0.01).

First, using the ex vivo mouse tissue spermatogenesis model and a conventional X-ray source, we tested radiation-induced effects on spermatogenesis. Figure 2 shows representative images of the expression of Acr-GFP in ex vivo testicular cultures after exposure to 0, 0.5, 1, 2, and 5 Gy conventional X-rays (150 keV). Our live-tissue imaging revealed a delay in dose-dependent peak expression was confirmed by observation 1 month after irradiation at dose of 0.5 Gy or greater. In addition, there was a significant dose-dependent decrease in the area of Acr-GFP following irradiation at dose of 2 Gy or greater. Thus, as previously reported [19], our results demonstrated the dose-dependent effects which may represent the clinical conditions of temporary infertility and permanent sterility.

### 3.2. Fractionated X-ray-Induced Effects on Spermatogenesis

**Figure 3:**
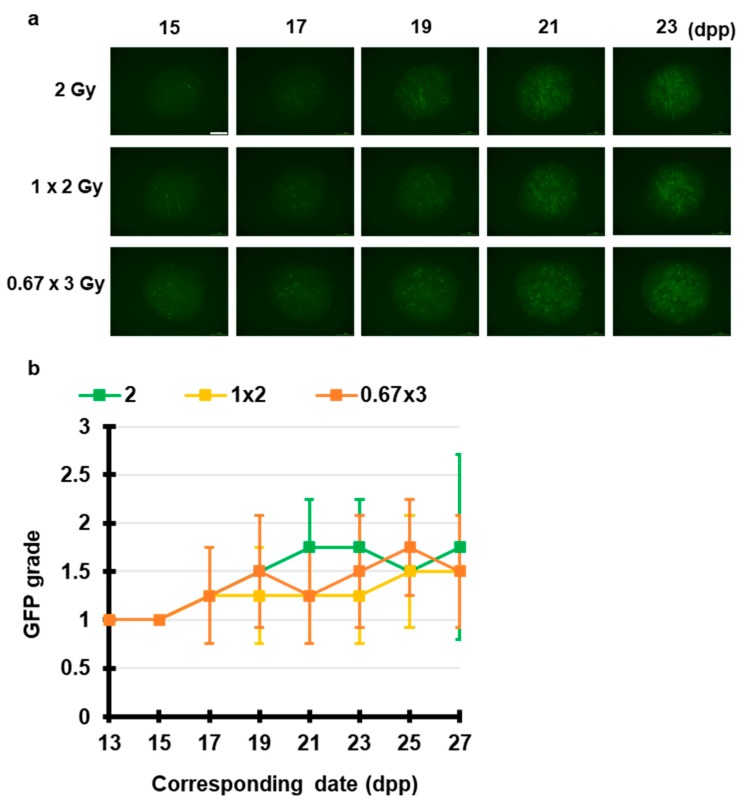
Chronological Acr-GFP expression changes after exposure to fractionated X-rays. (**a**) Representative images show Acr-GFP expression obtained at 15–23 dpp in single cultures following exposure to 2 Gy, 1 Gy × 2 (1 Gy per 24 h) and 0.67 Gy × 3 (0.67 Gy per 24 h) X-rays. Scale bar, 500 μm. (**b**) Evaluation of Acr-GFP expression from 13 to 27 dpp. Single tissue samples from each mouse testes were randomly assigned to experimental groups. At least four tissue samples each from different donor mice were used for each experiment; contaminated samples were excluded. Data show the mean Acr-GFP expression ± SD. There was no statistically significant difference between group means.

Next, we elucidated the fractionated radiation-induced effects on spermatogenesis. Figure 3 shows that 2 Gy, 1 Gy × 2 (1 Gy per 24 h) and 0.67 Gy × 3 (0.67 Gy per 24 h) induced Acr-GFP expression changes. Live-tissue imaging revealed that there was no significant difference in the Acr-GFP expression changes between the single and multiple fractionated irradiated tissues. Thus, temporally fractionated X-ray-induced effects could not be detected in this experimental system.

Previous clinical studies on radiotherapy-related male infertility showed that total doses to the testes exceeding 2.5 Gy delivered as multiple fractions generally produce permanent azoospermia, whereas for single-fraction exposures, the same effect would require doses over 6 Gy, indicating the temporally fractionated X-ray-induced effects on spermatogenesis [4,5]. The cause of this deviation would be the technical limitations associated with our method. For example, due to the lack of vascular and matrix structures in the ex vivo mouse testicular tissue culture, some aspects of the tissue microenvironment may be inaccurately captured. The relationship between these structures and radiation-induced signaling may differ from that in the actual in vivo scenario, where target tissues are surrounded by different cell types and interactions from multiple physiological systems.

### 3.3. Spatially Fractionated X-ray-Induced Effects on Spermatogenesis

**Figure 4:**
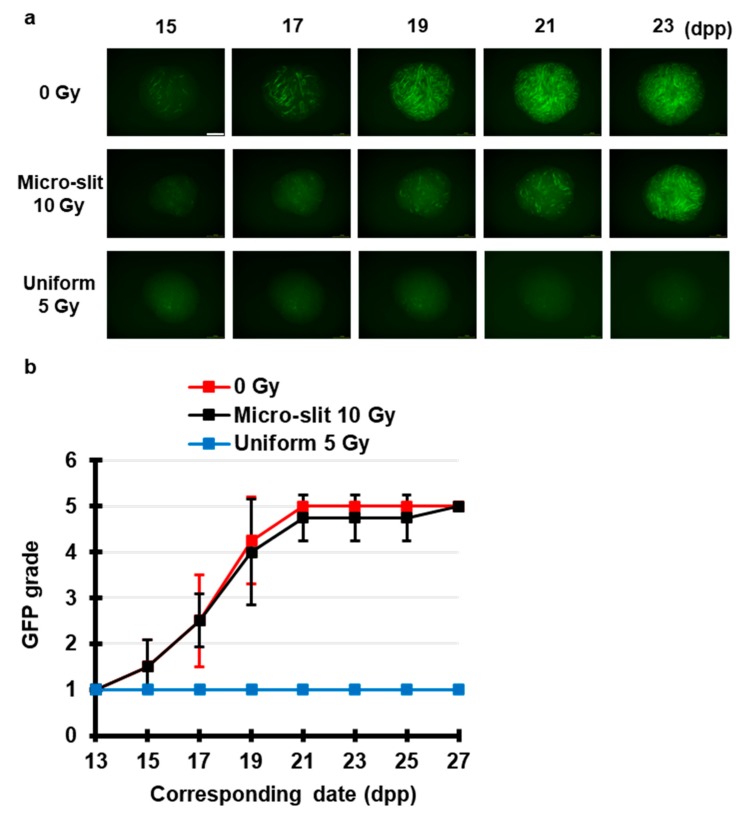
Chronological Acr-GFP expression changes after exposure to spatially fractionated X-rays. (**a**) Representative images show Acr-GFP expression changes in single cultures following 0 Gy (control), 10 Gy micro-slit (50%), and 5 Gy uniform (100%) X-ray irradiation from 15 to 23 dpp. Scale bar, 500 μm. (**b**) Chronological changes in Acr-GFP expression after micro-slit and uniform X-ray irradiation from 13 to 27 dpp. At least four tissue samples each from different donor mice were used for each experiment. Data represent the mean GFP expression ± SD. The Acr-GFP expression was significantly (*p* < 0.01) different between the uniform 5 Gy and the other group means.

To test our hypothesis, we investigated the TSE of spatially fractionated X-rays for maintaining spermatogenesis. Using Pb and acrylic plates, we constructed a 50% 300 μm-slit-modulated X-ray beams. As shown in Figure 4, live-tissue imaging revealed that a dose of 5 Gy applied uniformly almost completely obliterated the fluorescence signal of Acr-GFP from 15 to 27 dpp, whereas in the 50% 10 Gy micro-slit irradiation, the fluorescence signal remained similar to that of the non-irradiated controls, indicating the occurrence of a significant TSE for spermatogenesis.

To our knowledge, this study is the first to visualize the TSE for maintaining spermatogenesis following exposure to spatially fractionated conventional X-rays. As previously reported, high-precision MRT showed the TSE for spermatogenesis [16]. Taken together, our findings demonstrated that the TSE of spatially fractionated radiation responds to a wide range of energy of X-rays. This development is crucial for expanding the applicability of TSE in future clinical applications, particularly for the preservation of male fertility after radiotherapy.

### 3.4. X-ray-Induced Effects on Spermatogenesis in the Co-Cultured and Joined Tissues

**Figure 5:**
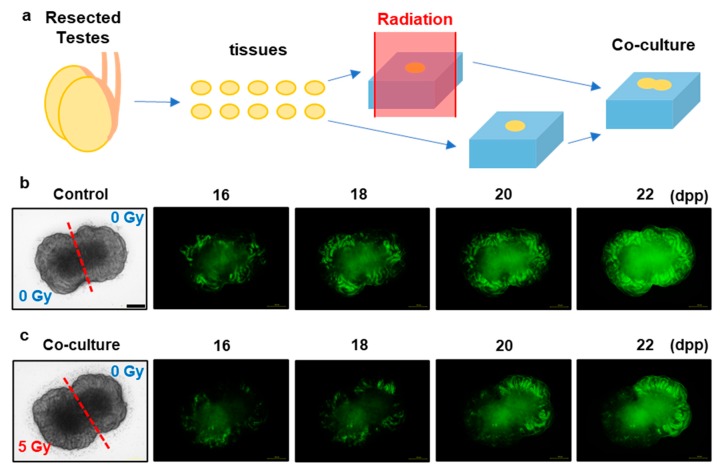
X-ray-induced effects on spermatogenesis in co-cultured and jointed tissue culture. (**a**) Schematic representation of the co-cultured and jointed tissue culture. The two testicular tissue samples were obtained from the same Acr-GFP transgenic mouse. One of these was exposed to 0 Gy (control) (**b**), and the other was exposed to 5 Gy X-rays (**c**). Representative images show Acr-GFP expression in the co-cultured and jointed tissue cultures from 16 to 22 dpp. The areas expressing Acr-GFP did not expand from the non-irradiated to the irradiated areas, although the central area of the co-cultured tissue showed autofluorescence. Therefore, the survival of spermatogonial cells in the non-irradiated areas and migration of these cells via the seminiferous tubules were essential for the TSE because the co-cultured and adjoined tissues had no individual seminiferous tubules connecting the irradiated and non-irradiated areas. Scale bar, 500 μm.

To investigate the TSE expansion process in the progress of spermatogenesis, we used the TSE in co-cultured and physically adjacent tissues (Figure 5). We directly placed the irradiated and non-irradiated tissues together; thus, the co-cultured and adjoined tissues had no individual seminiferous tubules connecting the irradiated and non-irradiated areas. In the co-cultured and adjoined tissues, no Acr-GFP expansion was observed from the non-irradiated to the irradiated areas, although the central area of the co-cultured tissue showed autofluorescence due to necrotic tissue damage. These findings indicate that the TSE in spermatogenesis requires two processes: survival of spermatogonial cells in non-irradiated areas and the migration of these cells via seminiferous tubules.

The International Commission on Radiological Protection (ICRP) considers the characteristics of stem cells and the niche as one of the most important factors for radiation-induced biological effects [25]. In fact, previous studies on seminiferous tubule repopulation following irradiation have indicated that spermatogonial stem cells begin colony formation soon after the destruction of differentiated germ cells [26]. Furthermore, male mouse germ cell transplantation was first performed by Ralph Brinster‘s research group in 1994 and resulted in donor cell spermatogenesis in the recipient testes, showing that the dynamics of spermatogonial stem cell (SSC) via seminiferous tubules are essential for maintaining spermatogenesis [27]. Recently, as previously reported [16], using synchrotron-generated X-ray microbeams and the ex vivo mouse spermatogenesis model, we also revealed that the survival and potential migration steps of the non-irradiated germ stem cells in the irradiated testes tissue are needed for effective TSE for spermatogenesis. These previous data support the present findings, namely, that the survival and migration of spermatogonial stem cells following exposure to spatially fractionated X-rays, not the radiation-induced intercellular signals from the non-irradiated to the irradiated areas in culture tissues, would be essential. Thus, further investigations of the control of stem cell migration should be performed in the field of stem cell biology and reproductive medicine.

## 4. Conclusions

In the present study, our findings revealed that the TSE of spatially fractionated radiation has considerable promise, as it responds to a wide range of energy of X-rays. In addition, we experimentally proposed a possible mechanism of TSE for spermatogenesis by which the migration of spermatogonial cells occurs from the non-irradiated areas to the irradiated ones via the seminiferous tubules. As a radiobiological approach for the preservation of male fertility, these contributions are crucial for expanding the applicability of this method. In future research, interdisciplinary investigations will be needed for the clinical application of our approach.
